# Evidence that low endocytic activity is not directly responsible for human serum resistance in the insect form of African trypanosomes

**DOI:** 10.1186/1756-0500-3-63

**Published:** 2010-03-05

**Authors:** Senthil KA Natesan, Lori Peacock, Ka Fai Leung, Wendy Gibson, Mark C Field

**Affiliations:** 1 Department of Pathology, Tennis Court Road, University of Cambridge, Cambridge, CB2 1QP, UK; 2 School of Biological Sciences, University of Bristol, Bristol, BS8 1UG, UK

## Abstract

**Background:**

In *Trypanosoma brucei*, the African trypanosome, endocytosis is developmentally regulated and substantially more active in all known mammalian infective stages. In both mammalian and insect stages endocytic activity is likely required for nutrient acquisition, but in bloodstream forms increased endocytosis is involved in recycling the variant surface glycoprotein and removing host immune factors from the surface. However, a rationale for low endocytic activity in insect stages has not been explored. Here we asked if endocytic down-regulation in the procyclic form was associated with resistance to innate trypanolytic immune factors in the blood meal or tsetse fly midgut.

**Findings:**

Using a well-characterized procyclic parasite with augmented endocytic flux mediated *via *TbRab5A overexpression, we found that insect stage parasites were able to grow both in the presence of trypanosome lytic factor (TLF) provided in human serum, and also in tsetse flies. Additionally, by placing blood stage parasites in restricted glucose medium, we observed that enlargement of the flagellar pocket, a key morphology associated with defective endocytosis, manifests in parallel with loss of cellular ATP levels.

**Conclusions:**

These observations suggest that a high rate of endocytosis *per se *is insufficient to render insect form parasites sensitive to TLF or tsetse-derived trypanocidal factors. However, the data do suggest that endocytosis is energetically burdensome, as endocytic activity is rapidly compromised on energy depletion in bloodstream stages. Hence an important aspect of endocytic modulation in the nutrient-poor tsetse midgut is likely energetic conservation.

## Introduction

*Trypanosoma brucei *is the causative agent of African sleeping sickness in humans and similar disease in sylvatic and domestic animals [1] and is transmitted by tsetse flies (genus *Glossina*). The life cycle involves several proliferative stages, two of which are readily grown in laboratory culture, bloodstream form (BSF) and procyclic form (PCF). The BSF is well known for its ability to evade the adaptive immune response of the mammalian host by antigenic variation. Endocytosis is developmentally regulated in trypanosomes and at least ten-fold up-regulated in BSFs compared to PCFs [2]. Nutrient uptake is a major function of endocytosis for all life stages, but the increased endocytic activity in BSFs potentially also offers protection against the mammalian immune system by efficient recycling of the variant surface glycoprotein (VSG) coat and rapid capping and internalization of anti-VSG antibody [3-5]. However, it is unclear if there is a similar defensive role associated with endocytosis in PCFs, or an adequate explanation for why the insect stages exhibits greatly reduced endocytic activity [6]. Recently it was proposed that this may protect against lytic factors in the blood meal, an attractive hypothesis given the presence of innate trypanolytic factors in human serum [7].

The principal lytic activity in human serum, trypanosome lytic factor (TLF), is associated with ApoLI, a high density lipoprotein (HDL) component, and very efficiently lyses *T. b. brucei *BSFs [7,8]. A haptoglobin-related protein in the HDL fraction binds to the trypanosome haptoglobin-hemoglobin receptor, resulting in uptake of HDL along with ApoLI, which finally leads to lysis of the parasite [9]. Protection from TLF killing is mediated by expression of serum resistance-associated antigen (SRA) protein, which binds ApoLI and prevents TLF entering the trypanosome lysosome [10]. SRA is expressed by human infective *T. b. rhodesiense*, but not *T. b. brucei *strains, which are fully sensitive to TLF lysis. The high rate of endocytosis may be a major contributor to TLF sensitivity and potential down-regulation of endocytic trafficking in the PCF could afford protection from TLF as PCFs do not express SRA [7,10]. This adaptation could also provide a rationale for rapid down-regulation of endocytic activity in PCF parasites that are exposed to insect lytic factors in the fly midgut [6]. Indeed, differentiation of BSF to PCF in the tsetse fly occurs within 24 hrs while components of the blood meal may persist for several days, meaning that PCFs are potentially exposed to both mammalian- and insect-derived trypanolytic factors.

To investigate further, we turned to a model previously established and well characterized in our laboratory. Over-expression of a Rab5AQL mutant isoform in *T. b. brucei *427 PCFs results in increased endocytosis; both fluid phase and receptor-mediated endocytosis in these cells is near-equivalent to BSFs [5,11]. Western blotting indicates that these Rab5AQL cells express levels of the clathrin heavy chain, the major structural mediator of endocytosis, also at levels that are similar to BSF cells [12]. Hence these cells represent a good model for analysis of the effects of augmented endocytosis on PCF sensitivity to innate lytic factors.

## Materials and methods

### Trypanosomes

Bloodstream and procyclic form *Trypanosoma brucei brucei *strain Lister 427 were grown as described [13,14]. Growth in modified media used glucose-free HMI-9 (Sigma) and dialyzed fetal bovine serum (FBS). Limiting glucose experiments were performed with glucose-free RPMI-1640 (11879-020, Gibco) supplemented with dialyzed FBS and specified concentrations of D-glucose or 2-deoxy-D-glucose. PCF lines Rab5AQL, Rab5AWT and Rab5ASN have been described and characterized in detail elsewhere [5,11,12].

### Tsetse experimental procedures

Male and female tsetse flies from the Bristol laboratory colony of *Glossina morsitans morsitans *were caged in groups of ~25 at 25°C and 70% relative humidity, and fed on sterile defibrinated horse blood *via *a silicone membrane. Flies were fed an infected meal for their first feed, 24-48 hours post-eclosion consisting of procyclic trypanosomes (approximately 10^7 ^cells/ml) and washed horse red blood cells resuspended in Hank's Balanced Salt Solution. At dissection, whole tsetse alimentary tracts, from the proventriculus to the rectum, were viewed as wet mounts in phosphate-buffered saline under bright field illumination. The relative number of trypanosomes was scored on a four point scale: negligible (1 - 5 trypanosomes), low (few trypanosomes scattered in midgut), moderate (some or all of midgut covered, individual trypanosomes discernible) and high (midgut densely filled, individual trypanosomes not discernible) [15]. Results from male and female flies were pooled and the chi-squared test used for comparing the infection rates between trypanosome cell lines.

### Immunofluorescence

Trypanosomes were harvested by centrifugation, washed with PBS and fixed with 4% PFA in ice-cold vPBS. Immunofluorescence was performed as described previously [16] with a few modifications. For presentation only, acquired gray scale images were false-colored, enhanced and assembled in Adobe Photoshop CS (Adobe Systems Inc).

### ATP measurement

ATP measurement in BSF parasites (5 × 10^6^/ml) was performed as described previously [17] with a few modifications. 2 × 10^7 ^BSF parasites were pelleted, washed in cold PBS and frozen in liquid nitrogen. The pellet was lysed by resuspending in 200 ul of ice-cold 0.9 M perchloric acid and neutralized by the addition of 100 ul of 2/0.5 M KOH/MOPS. Neutralization resulted in the formation of a white precipitate that was pelleted and the resulting supernatant was taken for ATP assay. ATP measurements were performed using an ATP bioluminescent assay kit (Sigma).

### Limiting glucose Concanavalin A (ConA) uptake assay

 BSF cells were grown in glucose-free RPMI-1640 media supplemented with 10% dialyzed FBS and 2.5 mM glucose for 12 h at 37°C. Cells were washed with glucose-free RPMI-1640 supplemented with 1% BSA and 1 mM glucose and subsequent assay was performed under these media conditions. FITC-ConA uptake assay was performed as described previously [18].

## Results and discussion

We set out to consider three inter-related questions. Firstly, does enhanced endocytosis lead to sensitivity of PCF forms to TLF or secondly, tsetse trypanolytic factors? Thirdly, could we obtain evidence that endocytosis is an energetically expensive activity?

**Figure 1:**
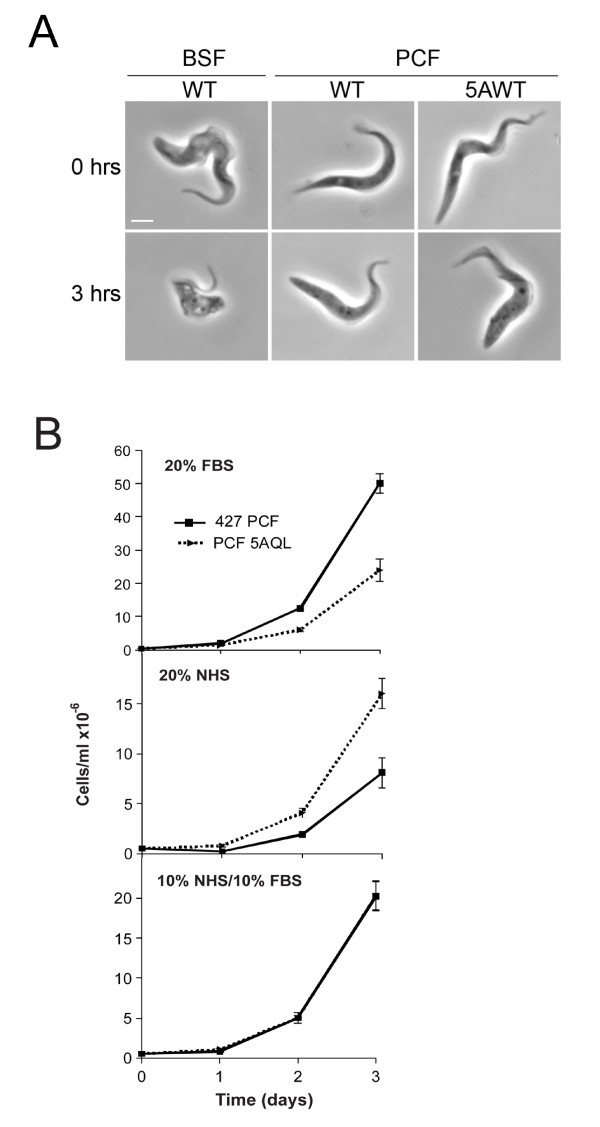
**High endocytic activity does not result in serum sensitivity in insect stage trypanosomes**. A. Phase contrast images of BSF and PCF cells taken at zero and three hours after incubation with 10% normal human serum (NHS). At zero hours both BSF and PCF parasites are intact while by three hours the BSF cells begin to exhibit vacuolation, while there is no change to the morphology of either wild type or Rab5AWT procyclic cells. Scale bar is 2 um. B. Growth curves for strain 427 procyclic line and PCF line over-expressing Rab5AQL (5AQL) cultured in different sera. The 5AQL line exhibits a rate of fluid phase and receptor-mediated endocytosis indistinguishable from bloodstream forms [11]. Untransformed 427 procyclics and the 5AQL line were cultured in SDM-79 medium supplemented with 20% FBS, 20% NHS or 10% FBS/10% NHS for several days under normal culture conditions. Parasite density was estimated daily with a haemocytometer. While the 427 PCF and 5AQL lines demonstrate differential growth in the single sera medium, in no case is there evidence for trypanocidal activity. The probability that preferential growth is due to limiting availability of a specific factor is supported by the identical growth observed when the sera are provided as a mixture. The data are from a single experiment done in duplicate, and are representative of several similar experiments. Error bars indicate standard deviation.

To address the first point, we examined survival of wild type and Rab5AQL PCF cells in the presence of heat-inactivated fetal bovine serum (FBS) and normal human serum (NHS) to assay sensitivity to serum trypanolytic factors, including TLF. As expected, in the presence of NHS, BSF cells rounded up with large vacuoles appearing very rapidly (Figure 1A), and in four hours all BSF cells were lysed. In the presence of 20% FBS, Rab5AQL cells showed slightly slower growth compared to wild type PCFs, as described previously [11]. However, culturing in the presence of 20% NHS resulted in faster growth for the Rab5AQL cells compared to wild type, and most importantly, with no evidence for trypanolytic activity. The differential growth rates of the two PCF lines may be ascribed to differential requirements for limiting factors in the respective sera, as a 1:1 mix of NHS and FBS resulted in indistinguishable growth rates (Figure 1B). In addition, the ability of NHS to lyse PCFs over-expressing Rab5AQL was further analyzed by the addition of fresh NHS (to a final concentration of 10%) to cultures every 24 hours to ensure that the lytic factor had not been inactivated. Again, all BSF cells were rapidly killed, but PCFs continued to proliferate, confirming that PCFs with enhanced endocytic activity are insensitive to TLF-mediated lysis (data not shown). Clearly, PCF resistance to TLF-mediated lysis does not require expression of SRA, as we used *T. b. brucei *427 which lacks the SRA gene, or require a low endocytic rate *per se*. This difference can also not be due to differential proliferation and cell cycle times as our PCF and BSF strains have similar doubling times at ~11 hours and ~8 hours respectively (Additional file 1). While the Rab5AQL mutant clearly cannot mimic the BSF endocytic pathway precisely and additional differences are present between the BSF and PCF endocytic system beyond gross activity, we can conclude that PCF resistance to TLF cannot be ascribed simply to low levels of endocytosis, and therefore additional factor(s) must operate.

**Figure 2:**
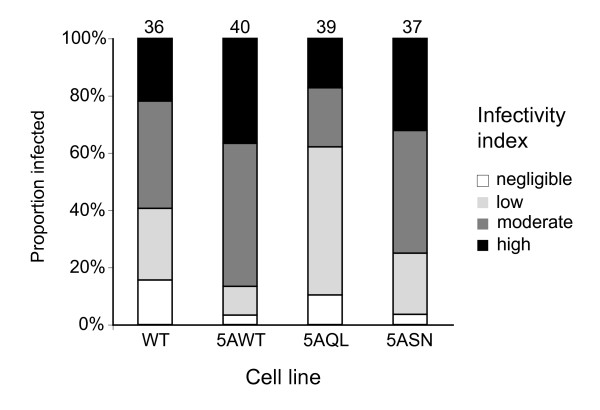
**PCF mutants with augmented endocytosis have no significant difference in tsetse midgut infectivity**. Tsetse flies were infected with wild type procyclic cells (PCF WT) and mutant PCF parasites 5AWT, 5AQL and 5ASN that have augmented endocytosis. The relative number of PCF parasites colonizing the tsetse midgut in each case was recorded at four or five days post-infection. See main text for statistical analysis of this dataset; the number of flies dissected for each strain is indicated above the relevant bar.

To directly determine if down-regulation of endocytosis in PCF is sufficient to avoid accumulation of lytic factors present in the tsetse fly midgut, we infected flies with PCF cells with augmented endocytic activity, over-expressing Rab5AWT, Rab5AQL and Rab5ASN [5,11]. Infection rates from pooled male and female tsetse flies provided no evidence that any over-expression cell line was significantly different from the wild type (P = 0.136-0.221, Chi-squared test). Additionally, there was no discernible difference between wild type and any over-expression line in the relative numbers of trypanosomes in fly midguts (Figure 2). Similar results of no major differences were obtained when this experiment was repeated with flies dissected 2-3 days (n = 58) or 8-9 days (n = 453) after infection (data not shown). Moreover, as growth of these mutant parasites with increased endocytic activity in the insect midgut was unhindered, tsetse factors did not inhibit cell division either. Hence reduced endocytosis in PCF is again unlikely to be specifically involved in avoiding fly immune factors in the midgut. Therefore, we conclude that reduced endocytosis in PCF is not sufficient for avoiding uptake of either TLF or insect immune factors.

Thirdly, to investigate the contribution of energy metabolism to endocytosis, BSF parasites were cultured in a range of concentrations of D-glucose and 2-deoxy-D-glucose to limit the primary carbon source for ATP synthesis. We monitored the emergence of a BigEye flagellar pocket phenotype as evidence for decreased endocytosis [19], and motility of the cells as evidence of the continued presence of ATP. Finally we also directly assayed ATP level. Initially we added 2-deoxy-D-glucose to HMI-9 medium, which inhibits uptake of glucose *via *the glucose transporter, and to provoke a complete block to ongoing glycolysis [20]. At ranges of 0.1-25 mM the parasites immediately ceased moving and had no change to their morphology, suggesting a catastrophic loss of ATP as expected (Table 1). Therefore complete de-energizing does not result in a BigEye morphology.

**Table 1:** Effect of glucose deprivation on motility and morphology in bloodstream trypanosomes.

Treatment	Conc (mM)	15 min		2 h		6 h		8 h		12 h	
	
	morp		mot	morp	mot	morp	mot	morp	mot	morp	mot
Control	25	100%	100%	100%	100%	100%	100%	100%	100%	100%	100%

2-deoxy-D-glucose	0.1	Dead	Dead	NA	NA	NA	NA	NA	NA	NA	NA
	
	1.0	Dead	Dead	NA	NA	NA	NA	NA	NA	NA	NA
	
	10	Dead	Dead	NA	NA	NA	NA	NA	NA	NA	NA

D-Glucose	0.0	Dead	Dead	NA	NA	NA	NA	NA	NA	NA	NA
	
	0.1	Dead	Dead	NA	NA	NA	NA	NA	NA	NA	NA
	
	1.0	100%	50%	Dead	Dead	NA	NA	NA	NA	NA	NA
	
	5.0	100%	100%	90%	100%	65%	80%	55%	65%	20%	35%
	
	10	100%	100%	100%	100%	100%	100%	80%	90%	50%	50%

Log-phase trypanosomes were harvested, cells washed in PBS and resuspended in HMI-9 medium supplemented with 2-deoxy-D-glucose, in standard formulation HMI-9 (25 mM) or glucose free RPMI-1640 supplemented with D-glucose as indicated. All media also contained dialyzed 10% fetal bovine serum. Cells were incubated at 37°C and observed under phase contrast at intervals. Aliquots were withdrawn at various times and mounted on pre-warmed microscope slides. Normal cells were scored as a length:breadth ratio of >5:1 while rounded cells scored with a ratio of 2:1 or less. "morp"; percent of cells retaining normal morphology. Abnormalities includes rounded cells or emergence of a BigEye flagellar pocket. "mot"; motility; cells were scored as motile if there was evidence of cell movement, including flagellar twitching. NA; not analyzed.

**Figure 3:**
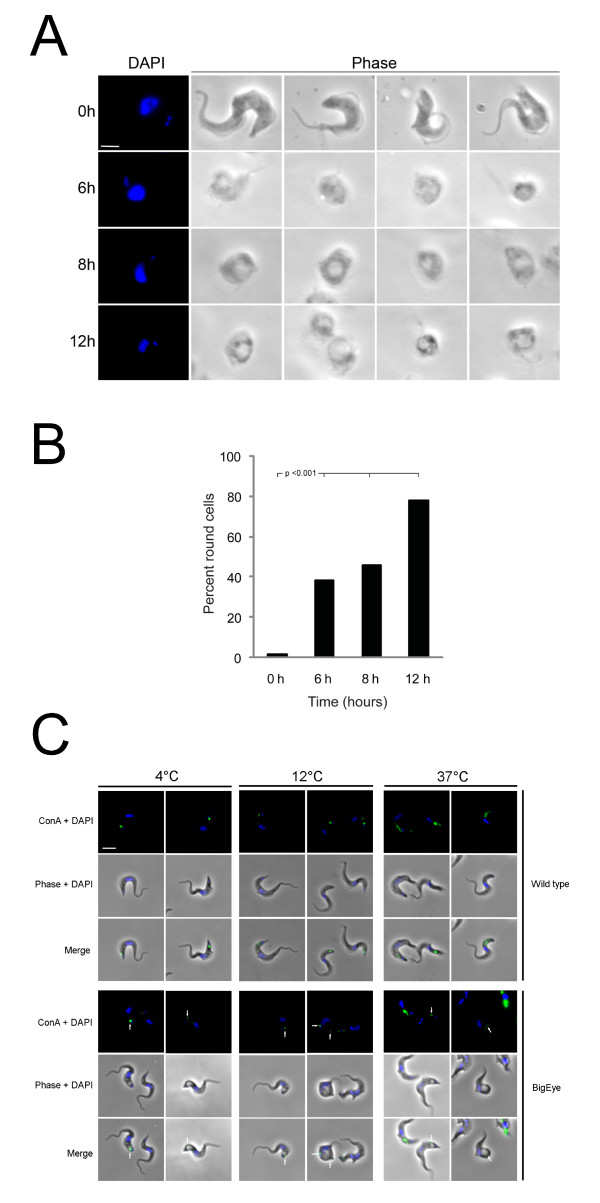
**Reduced glucose levels in growth media for *T. brucei *leads to the appearance of the 'BigEye' phenotype and is lethal to BSF parasites**. A. *T. brucei *BSF parasites were cultured in RPMI-1640 media containing 5 mM D-glucose and dialyzed (glucose-free) 10% FBS. At time zero the parasites have normal phenotype, but after six hours the cells became rounded. From six to twelve hours the flagellar pocket increased in diameter. Scale bar 2 um. B. The number of parasites with BigEye phenotype increases with time when grown in reduced glucose conditions. Cultures in glucose-replete medium contain essentially no BigEye cells, but by contrast, in reduced glucose medium (5 mM) at six hours rounded cells contribute 35% of the population, which increases to 45% and 80% by eight and twelve hours respectively. Statistical support for a significant increase in rounded cells is indicated using a chi-squared test. Note that at least 200 cells were examined for each condition, and all treated conditions are highly significantly different from the control (p < 0.001). C. Limiting glucose levels affects endocytosis in BSF parasites. ConA uptake was observed following growth under limiting glucose conditions. Cells in complete medium showed normal morphology while glucose-starved cells displayed the BigEye phenotype as observed by phase contrast. Location of FITC-ConA (green) in fixed cells co-stained with DAPI (blue) was observed by fluorescence microscopy at 4°C (flagellar pocket), 12°C (early endosomes) and 37°C (lysosomes). Arrows indicate ConA trapped at the flagellar pocket of BigEye cells. Scale bar is 2 um.

Parasites cultured in HMI-9 medium with 0.1 mM or no D-glucose again ceased movement instantly. However, at 5.0 mM D-glucose, BSF parasites were able to survive for a considerable period (Table 1). At the start of the experiment essentially no cells exhibited the enlarged flagellar pocket morphology, but by six hours 35% of the parasites had rounded morphology with an enlarged flagellar pocket and by twelve hours >80% of the parasites had this morphology (Table 1 and Figure 3). The flagellum of these round cells continued to twitch, indicating they retained sufficient ATP to power residual motility. In 10 mM D-glucose the appearance of rounded cells and cell death was delayed, with only 50% rounded cells after twelve hours (Table 1). Although the rounded cells were motile, it is clear that motility was less than normal, indicating that effects on cellular processes in addition to endocytosis were severe.

To confirm that limiting glucose affects endocytosis, we monitored uptake of Concanavalin A (ConA) lectin on cells that had been starved of glucose. In control cells, ConA labels the flagellar pocket at 4°C due to inhibition of endocytosis (Figure 3C, top panel). At 12°C, endocytosis takes place, with ConA being found on early endosomes, while at 37°C ConA reaches the lysosomes [18]. By contrast, ConA is restricted to the edge of the enlarged flagellar pocket in cells grown under limiting glucose, even at 37°C. This is identical to the phenotype observed when endocytosis is blocked directly by clathrin knockdown [19], confirming inhibition of endocytosis under these conditions (Figure 3C, bottom panel). These observations suggest a high energy requirement for maintaining the bloodstream endocytic machinery as an imbalance in endocytic and exocytic flux emerges rapidly on reduced glucose availability; specifically the BigEye morphology emerges in cells where exocytosis remains functional, but endocytic activity is compromised. In addition several other cellular functions are likely compromised by decreased ATP production, amongst them motility. Clearly the energy needs for endocytosis and other functions can be met in the nutrient-rich mammalian bloodstream.

**Figure 4:**
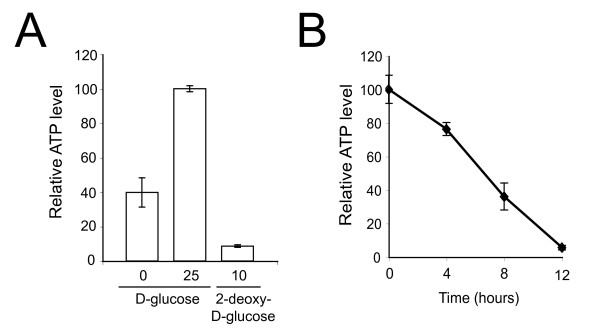
***T. brucei *BSF parasites cultured under low glucose conditions possess reduced ATP levels**. A. *T. brucei *BSF parasites were either cultured in RPMI-1640 media containing 0 mM or 25 mM D-glucose or 10 mM 2-deoxy-D-glucose, all supplemented with dialyzed (glucose-free) 10% FBS. A reduction in ATP was observed in parasites cultured in either 0 mM D-glucose or 10 mM 2-deoxy-D-glucose. Data are normalized to 100% for 25 mM D-glucose and are the mean of three independent assays. Error bars show the standard deviation. B. *T. brucei *BSF parasites were cultured in RPMI-1640 media containing 5 mM D-glucose and dialyzed (glucose-free) 10% FBS for twelve hours and ATP levels measured at intervals. Data are the mean of three independent determinations, and error bars show the standard deviation.

We confirmed that culturing parasites under low glucose concentrations or with 2-deoxy-D-glucose indeed results in reduced ATP production. BSF parasites cultured without D-glucose show rapid reduction in ATP level to 40% of that in parasites grown in 25 mM D-glucose after five minutes in low glucose medium (Figure 4A). However, ATP decreased to 10% of the normal level in parasites cultured with 10 mM 2-deoxy-D-glucose, again within five minutes exposure (Figure 4A). Further, we measured the ATP levels in parasites cultured with 5.0 mM D-glucose over a period of 12 hours. By four hours ATP levels were 75-80%, by eight hours 25-45% and less than 10% after tweve hours (Figure 4B). We infer that ATP generation is reduced in parasites cultured under limiting glucose, depleting the energy required for endocytosis, in turn resulting in enlargement of the flagellar pocket.

Trypanosomes encounter major changes to the levels and type of nutrients and oxygen tension when alternating between mammalian and insect hosts. They respond to this by differentiating their primary metabolism and by remodelling the cell surface [21]. BSFs express enzymes for utilizing primarily glucose as substrate to produce energy *via *glycolysis in the glycosome and are entirely dependent on substrate level phosphorylation for energy production [21-23]. No additional energy is generated by oxidative phosphorylation, but given the glucose-rich environment a low yield production of ATP is clearly sufficient. However, in the tsetse midgut trypanosomes are exposed to diminishing glucose concentrations as the blood meal is digested and subsequently depend on proline as their primary energy source [24]. In PCFs the mitochondrion is well-developed, with cytochrome-mediated electron transport chains and an active Krebs cycle that contribute to a greater ATP yield [22,25-27]; presumably this is to facilitate more efficient use of limited energy sources.

Hence it is possible that an additional strategy employed by the PCF is to down-regulate endocytosis, as a high level is clearly not required during development in the insect host [6]. This is not to suggest that additional cellular functions are not also energy demanding, but that high endocytosis is apparently one function that can be dispensed with in the tsetse fly. Significantly, metacyclic trypanosomes re-activate their endocytic apparatus and remodel their mitochondria and glycosomes so that they are pre-adapted to life in the mammalian bloodstream [6,28]. However, metacyclics are small cells, do not divide until they arrive in the vertebrate host and have sluggish motility; their energy requirements are presumably modest, allowing prolonged survival.

In conclusion, we evaluated sensitivity to mammalian and insect-derived trypanolytic factors in cells with augmented endocytic flux. Using well-characterized PCF mutants over-expressing Rab5A isoforms, with augmented endocytic activity similar to bloodstream stages [5,11,12], we find no evidence endocytic down-regulation is essential for avoiding uptake of noxious components from mammalian serum or the tsetse midgut. The insect stage trypanosome likely has additional mechanisms to avoid lysis distinct from the SRA gene product, including the developmental regulation of the TbHbHp receptor [7,9]. Therefore it remains most probable that additional differences between PCF and BSF cells, unaltered by Rab5A-mediated endocytosis augmentation, play important roles in TLF resistance in PCFs.

We earlier argued that modulation of endocytic activity does not reflect a simple nutrient uptake requirement, as in culture both PCF and BSF have similar generation times, and PCF endocytosis is likely sufficient for uptake of nutrients requiring receptor-mediated mechanisms [6]. Low molecular weight metabolites, including hexoses and amino acids, gain entry to the cell *via *plasma membrane transporters, and again there is no need to invoke an endocytic aspect. Rather, we suggest that modulation contributes to energy conservation, as one first clear manifestation of ATP depletion is an enlarged flagellar pocket and a loss of endocytosis. Additional processes almost certainly are affected by decreased ATP levels, and provision for these processes probably also contribute to the adaptive response of trypanosome to conditions within the tsetste fly. Energetic considerations, specifically a less rich nutrient source, are consistent with the tsetse being a more challenging environment for the trypanosome.

## Competing interests

The authors declare that they have no competing interests.

## Authors' contributions

MCF and WG conceived the research. SKAN, LP and KFL performed the work. All authors contributed to data analysis and writing of the paper.

## Supplementary Material

Additional file 1 **Proliferation data for procyclic and bloodstream form *T. brucei *in culture**. Raw data and plots for cell numbers for procyclic and bloodstream form *T. brucei *in culture in SDM79 and HMI-9 media respectively. Click here for file
