# Supplementary material for: Evidence that low endocytic activity is not directly responsible for human serum resistance in the insect form of African trypanosomes
Source: BMC Res Notes. 2010 Mar 5;3:63. doi: 10.1186/1756-0500-3-63 (PMC2848055; doi:10.1186/1756-0500-3-63)

Day	427 BSF (1)	Cell number		427 BSF (2)	Cell number
0	0.10	0.10	0	0.10	0.10
1	0.92	9.20	1	1.10	11.00
1	0.10		1	0.10	
2	1.05	96.60	2	1.34	147.40
2	0.10		2	0.10	
3	1.06	1023.96	3	0.72	1061.28
3	0.10		3	0.10	
4	1.33	13618.67	4	1.31	13902.77
4	0.10		4	0.10	
5	0.75	102140.01	5	1.01	140417.96
5	0.10		5	0.10	
6	1.16	1184824.12	6	1.22	1713099.07
6	0.10		6	0.10	
7	0.98	11611276.34	7	1.05	17987540.27

	Cell number	Cell number	Average	log	STDEV
0	0.10	0.10	0.10	-1	0.00
1	9.30	11.10	10.20	1.008600172	1.27
2	105.90	158.50	132.20	2.121231455	37.19
3	1129.86	1219.78	1174.82	3.069971331	63.58
4	14748.53	15122.55	14935.54	4.174220871	264.47
5	116888.54	155540.50	136214.52	5.134223409	27331.07
6	1301712.65	1868639.58	1585176.12	6.20007752	400877.87
7	12912988.99	19856179.84	16384584.42	7.21443543	4909577.34

	427 PCF (1)	Cell number		427 PCF (2)	Cell number
0	0.10	0.10	0	0.10	0.10
1	1.28	12.80	1	1.13	11.30
2	5.71	57.10	2	5.50	55.00
2	1.00		2	1.00	
3	4.49	256.38	3	5.84	321.20
3	0.10		3	0.10	
5	2.48	6358.20	5	2.75	8833.00
5	1.00		5	1.00	
6	4.80	30519.36	6	5.63	49729.79
6	1.00		6	1.00	
7	4.83	147408.49	7	4.60	228757.03

	Cell number	Cell number	Average	log	STDEV
0	0.10	0.10	0.10	-1	0.00
1	12.90	11.40	12.15	1.084576278	1.06
2	70.00	66.40	68.20	1.833784375	2.55
3	326.38	387.60	356.99	2.552655443	43.29
5	6684.58	9220.60	7952.59	3.900508544	1793.24
6	37203.93	58950.39	48077.16	4.681938825	15377.07
7	184612.42	287707.42	236159.92	5.373206201	72899.17

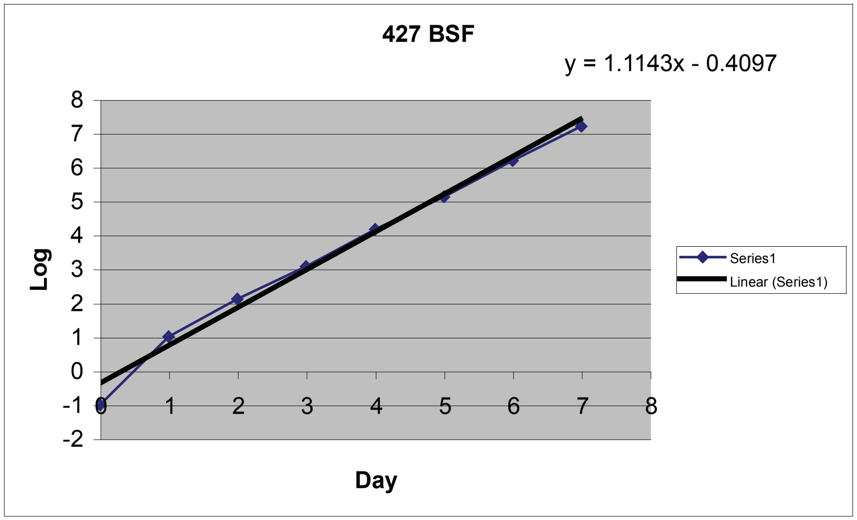

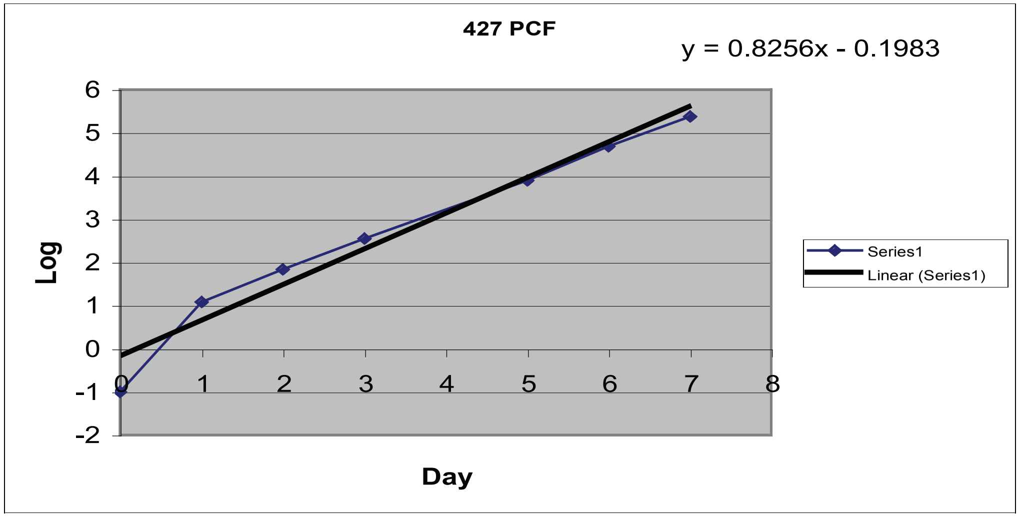

Supplement: Additional file 1 — Proliferation data for procyclic and bloodstream form T. brucei in culture. Raw data and plots for cell numbers for procyclic and bloodstream form T. brucei in culture in SDM79 and HMI-9 media respectively. [file 1756-0500-3-63-S1.PDF]
